# Efficacy and safety of nivolumab for advanced gastric cancer patients with poor performance statuses

**DOI:** 10.1186/s12885-020-07176-7

**Published:** 2020-07-22

**Authors:** Toshihiko Matsumoto, Yosuke Yamamoto, Yusuke Kuriona, Ukyo Okazaki, Shogo Kimura, Kou Miura, Takao Tsuduki, Takanori Watanabe, Yusuke Mastumoto, Masahiro Takatani

**Affiliations:** 1 Department of Internal Medicine, Himeji Red Cross Hospital, 1-12-1, Shimoteno, Himeji, Hyogo 6708540 Japan; 2 Department of Clinical Oncology, Kobe city medical center general hospital, 2-1-1, minatojimaminamimachi, Kobe, Hyogo 6500047 Japan; 3 Department of Surgery, Himeji Red Cross Hospital, 1-12-1, Shimoteno, Himeji, Hyogo 6708540 Japan

**Keywords:** Gastric cancer, Nivolumab, Treatment failure

## Abstract

**Background:**

Nivolumab has changed the treatment of advanced gastric cancer (AGC). Nivolumab shows better outcomes compared to best supportive care among AGC patients who received at least two prior regimens. However, there are no reliable data regarding AGC patients with poor performance status (PS) who received nivolumab. We investigated the efficacy and safety of nivolumab among AGC patients with poor PS.

**Methods:**

We retrospectively collected clinicopathologic data from patients with AGC who underwent nivolumab monotherapy at our institution from October 2017 to June 2019.

**Results:**

Forty-nine AGC patients who received nivolumab were assessed. Twenty-seven patients had PS 0 or 1 (Good group) and 22 had PS 2 or 3 (Poor group). The median progression-free survival and overall survival durations were 2.0 and 6.0 months in the Good group, respectively, and 1.2 and 2.8 months in the Poor group, respectively. The overall survival was significantly shorter in the Poor group (6.0 vs 2.8 months, *p* = 0.0255). The disease control rates were 23 and 9% in the Good and Poor groups, respectively. Thirty-three percent of patients experienced immune-related adverse events in the Good group, and 18% in the Poor group.

**Conclusion:**

Nivolumab is feasible but insufficient as third- or later-line treatment for AGC patients with poor PS.

## Background

Gastric cancer is one of the most common forms of cancer. Its incidence is the fifth highest among cancers worldwide and it is the third commonest cause of deaths due to cancer [1]. Globally, systemic chemotherapy is the standard treatment for unresectable and metastatic gastric cancer. Combinations of a fluoropyrimidine, platinum agent, and taxane are standard first-line chemotherapeutic regimens for patients with human epidermal growth factor receptor 2 (HER2)-negative advanced gastric cancer (AGC). In HER2-positive AGC, a fluoropyrimidine, platinum agent, and trastuzumab (an anti-HER2 antibody) are standard first-line chemotherapeutic regimens [2–4].

Recently, blockade of immune checkpoint molecules with monoclonal antibodies has demonstrated promising efficacy for AGC. Nivolumab is a fully human IgG4 monoclonal antibody inhibitor of programmed death-1 (PD-1). The phase III trial of nivolumab (ATTRACTION-2) for patients with AGC after two or more previous lines of chemotherapy in Japan, South Korea and Taiwan showed a significant survival benefit, and nivolumab has been approved for AGC in Japan [5]. Another PD-1 antibody (pembrolizumab) also showed clinical benefit for PD-L1 positive AGC in phase 2 and 3 trials [6, 7].

In those clinical trials, no AGC patients had poor general conditions, such as Eastern Cooperative Oncology Group (ECOG) performance status (PS) 2–4. In the ATTRACTION-2 trial, 29% of patients had PS 0 and 71% had PS 1. In the real world in our institution, not only AGC patients in good condition but also those in poor condition receive nivolumab after two or more previous line chemotherapeutic regimens. Among AGC patients in poor condition, it remains unclear whether nivolumab has sufficient efficacy and tolerable toxicity. The aim of the present study was to assess the safety and efficacy of nivolumab among AGC patients with poor conditions and investigate their prognostic factors.

## Methods

### Patients

This study was single institution study. The subjects were patients with AGC treated with nivolumab between October 2017 and June 2019 at the Himeji Red Cross Hospital, Hyogo, Japan. All data were collected retrospectively from electronic medical records. All procedures were performed in accordance with institutional and national standards on human experimentation, as confirmed by the ethics committee of Himeji Red Cross Hospital, and with the Declaration of Helsinki of 1964 and its later amendments.

The inclusion criteria were as follows: (1) unresectable gastric cancer, (2) histologically proven gastric carcinoma, (3) refractory or intolerant to at least 2 regimens, and (4) no prior administration of immune checkpoint inhibitors. The study protocol was approved by the Institutional Review Board of the Himeji Red Cross Hospital.

### Treatment

The patients received nivolumab 3 mg/kg infusion every 2 weeks until disease progression or intolerance (240 mg/kg since August 2018).

### Evaluation and statistical analysis

ECOG perfomance score was defined by the clinical oncologists and chemotherapeutic nurses. Tumour response was evaluated according to the Response Evaluation Criteria in Solid Tumors (RECIST) version 1.1. Toxicity was assessed using the Common Terminology Criteria for Adverse Events (CTCAE) version 4.1. Overall survival (OS) was assessed from the date of initiation of treatment with nivolumab until death. Patients who were alive or for whom data were missing at the data cut-off point were censored. Progression-free survival (PFS) was assessed from the date of initiation of treatment with nivolumab until disease progression was confirmed. Patients for whom there was no information regarding tumour progression were treated as censored cases. OS and PFS were estimated using the Kaplan–Meier method. Statistical analyses were performed using JMP version 12 (SAS Institute Inc., Cary, NC, USA).

### Prognostic factors

Among the study subjects, we assessed the Japan Clinical Oncology Group (JCOG) index, Royal Marsden Hospital (RMH) index, and modified Glasgow prognostic score (mGPS). The JCOG index comprised four risk factors: ECOG PS ≥1, number of metastatic sites ≥2, no prior gastrectomy, and serum alkaline phosphatase (ALP) level (normal range:). Based on these factors, the risks among patients were classified as follows: good risk (0–1 factor), moderate risk (2–3 factors), and poor risk (4 factors) [8]. The RMH index consists of the following four independent risk factors for survival: PS ≥2, liver metastasis, peritoneal metastasis, and serum ALP concentration ≥ 100 U/L. Patients were classified into the following three groups according to the number of risk factors: low risk (no risk factors), moderate risk (1 or 2 risk factors), and high risk (3 or 4 risk factors) [9]. The mGPS was assessed based on elevated serum C-reactive protein (CRP) concentration and hypoalbuminemia. Patients with an elevated serum CRP concentration (> 10 mg/L) and hypoalbuminemia (serum albumin concentration < 35 g/L) were allocated a score of 2. Patients with an elevated serum CRP concentration (> 10 mg/L) alone received a score of 1, and those with a normal CRP concentration (≤10 mg/L) and any albumin concentration received a score of 0 [10].

## Results

Between October 2017 and June 2019, 49 patients received nivolumab after failure of at least 2 regimens. Their characteristics are shown in Table 1. The median age was 67 (range: 42–83) years and a majority was male (80%). Twenty-seven patients were allocated a PS 0 or 1 (Good group) and 22 patients were allocated a PS 2 or 3 (Poor group). Twenty-four patients (49%) had a diffuse type histology and 10 patients (20%) had HER2 positive disease. Micro satellite instability was not tested in all patients. Thirty-three patients (67%) had peritoneal dissemination and 19 (39%) had liver metastasis. All patients received regimens containing 5-fluorouracil, 41 (84%) received platinum-containing regimens, 46 (94%) received taxane, 40 (82%) received ramucirumab, and 11 (22%) received a CPT-11 containing regimen.

**Table 1:** Background characteristics of study participants

		All (***n*** = 49), n (%)	Good PS (***n*** = 27), n (%)	Poor PS (***n*** = 22), n (%)
Age, yrs	Median (range)	67 (42–83)	69 (48–72)	66.5 (42–83)
Sex	Male	39 (80)	9 (33)	19 (86)
PS	0	5 (10)	5 (19)	0
	1	22 (45)	22 (81)	0
	2	14 (29)	0	14 (64)
	3	8 (16)	0	8 (36)
Histology	Diffuse type	24 (49)	12 (44)	12 (55)
	Intestinal type	24 (49)	15 (56)	9 (41)
HER2 status	Positive	10 (20)	5 (19)	5 (23)
Prior gastrectomy	Yes	29 (59)	15 (56)	14 (55)
Number of metastatic sites	≥2	27 (55)	15 (56)	12 (55)
Liver metastasis	Yes	19 (39)	14 (56)	5 (23)
Peritoneal dissemination	Yes	33 (67)	17 (63)	16 (73)
Ascites	Yes	27 (55)	11 (41)	16 (73)
Number of prior regimens	2	35 (71)	19 (70)	16 (73)
	3	10 (20)	7 (26)	3 (14)
	> 3	4 (8)	2 (4)	2 (9)
Prior 5-FU	Yes	49 (100)	27 (100)	22 (100)
Prior platinum	Yes	41 (84)	22 (81)	19 (86)
Prior taxane	Yes	46 (94)	25 (93)	21 (95)
Prior irinotecan	Yes	11 (22)	8 (30)	3 (14)
Prior ramucirumab	Yes	40 (82)	23 (85)	17 (77)

*PS* performance status, *HER2* human epidermal growth factor receptor, *5-FU* 5-fluorouracil

### Efficacy

**Fig. 1:**
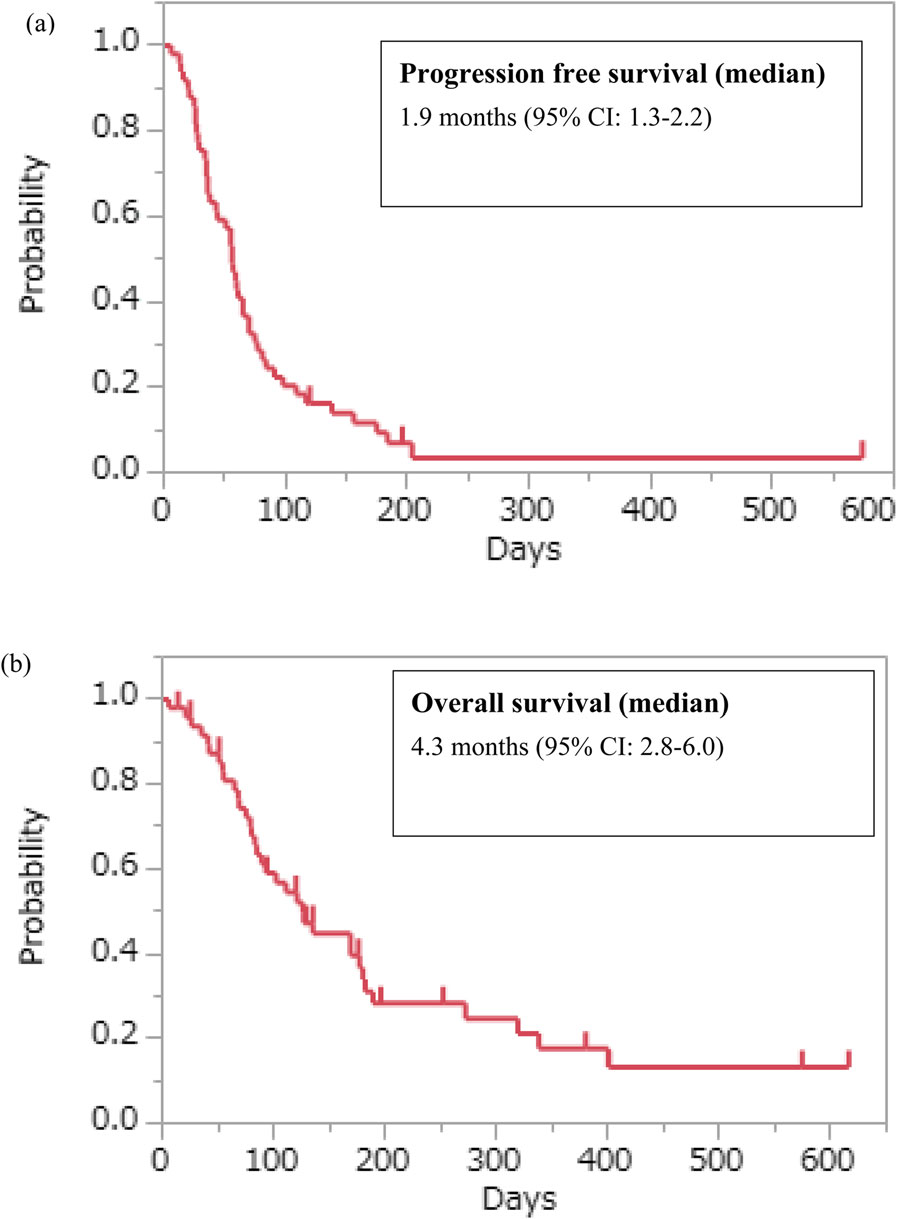
Kaplan–Meier plots of **a** progression-free survival (PFS) and **b** overall survival (OS) among study participants

**Fig. 2:**
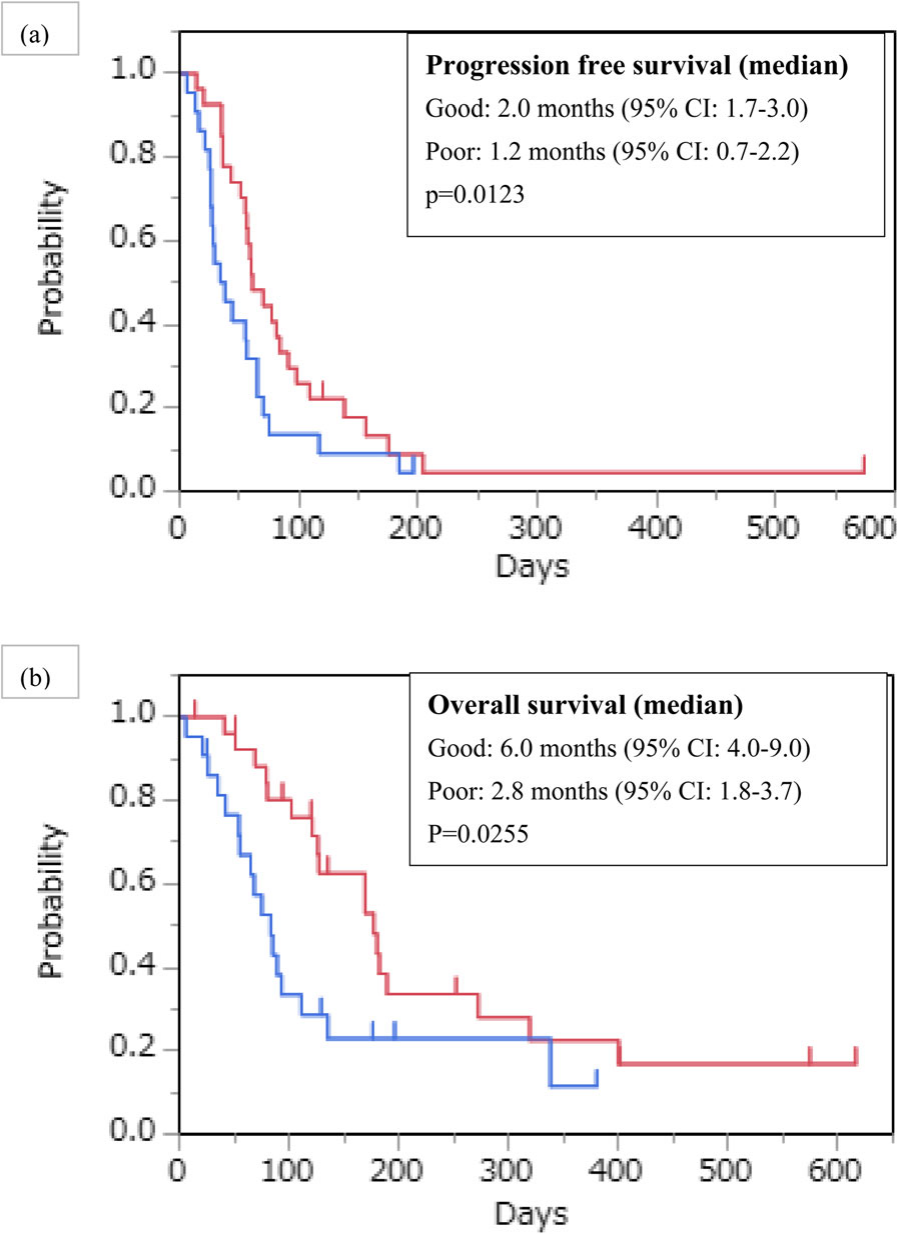
Kaplan–Meier plots of **a** progression-free survival (PFS) and **b** overall survival (OS) among study participants. Red line: Good group, Blue line: Poor group

Forty-seven patients with measurable lesions were evaluated for tumour response. A total of 6% of patients achieved a partial response, and 17% of patients showed stable disease, resulting in a response rate (RR) of 6% and a disease control rate (DCR) of 23% (Table 2). The median follow-up time was 155 days among censored cases. The median PFS was 1.9 months (95% confidence interval [CI], 1.3–2.2), and the median OS was 4.3 months (95% CI, 2.8–6) (Fig. 1). In the Good group, the RR was 8%, the DCR was 27%, the median PFS was 2.0 months (95% CI, 1.7–3.0), and the OS was 6.0 months (95% CI, 4.0–9.0). In the Poor group, the RR was 5%, the DCR was 19%, the median PFS was 1.2 months (95% CI, 0.7–2.2), and the OS was 2.8 months (95% CI, 1.8–3.7) (Figs. 2). There were significant differences in PFS and OS between the Good and Poor groups (Fig. 2). In the Poor group, only 1 patient achieved PR but with poor PS due to complications such as brain infarction.

**Table 2:** Responses among patients with measurable lesions

	All (***n*** = 49)	Good (***n*** = 27)	Poor (***n*** = 22)
CR	0	0	0
PR	3	2	1
SD	8	5	3
PD	36	19	17
NE	2	1	1
RR (%)	6%	8%	5%
DCR (%)	23%	27%	19%

*CR* complete response, *PR* partial response, *SD* stable disease, *PD* progressive disease, *NE* not evaluable, *RR* response rate, *DCR* disease control rate (CR + PR + SD)

### Safety

Adverse events among study participants are shown in Table 3. Thirty-three percent of patients experienced immune-related adverse events (irAE) in the Good group, and 18% in the Poor group. There was no significant difference in safety between the Good and Poor groups. One patient died due to grade 5 colitis in the Poor group. There was no significant difference between the Good and Poor groups regarding safety characteristics.

**Table 3:** Distribution of adverse events among study participants

	All (***n*** = 49), n (%)			Good (***n*** = 27), n (%)			Poor (***n*** = 22), n (%)		
	All	G 1/2	≥G 3	All	G 1/2	≥G 3	All	G 1/2	≥G 3
Fatigue	4 (8)	4 (8)	0	2 (7)	2 (7)	0	2 (9)	2 (9)	0
Pruritis	3 (6)	3 (6)	0	3 (11)	3 (11)	0	0	0	0
Diarrhoea	6 (12)	3 (6)	3 (6)	2 (7)	0	2 (7)	4 (18)	3 (14)	1 (5)
Anorexia	2 (4)	2 (4)	0	1 (4)	1 (4)	0	1 (5)	1 (5)	0
Hypothyroidism	2 (4)	2 (4)	0	1 (4)	1 (4)	0	1 (5)	1 (5)	0
Rash	3 (6)	2 (4)	1 (2)	3 (11)	2 (7)	1 (4)	0	0	0
Hyperglycaemia	1 (2)	0	1 (2)	1 (4)	0	1 (4)	0	0	0
Liver dysfunction	1 (2)	1 (2)	0	0	0	0	1 (5)	1 (5)	0
Adrenal insufficiency	2 (4)	2 (4)	0	2 (7)	2 (7)	0	0	0	0
Stomatitis	1 (2)	1 (2)	0	1 (4)	1 (4)	0	0	0	0
Fever	1 (2)	1 (2)	0	1 (4)	1 (4)	0	0	0	0

## Discussion

In our study, nivolumab showed poor survival outcomes regarding poor PS among gastric cancer patients. In the ATTRACTION-2 trial, poor PS (PS 1), low serum sodium concentration, high neutrophil-to-lymphocyte ratio, and no prior ramucirumab suggested poor prognosis among AGC patients who received nivolumab [5]. The results of subgroup analysis in phase 2 and 3 trials of pembrolizumab showed that better PS was associated with a higher RR and longer OS [6, 7]. However, no patients had PS 2–4 in these trials. Spigel DR et al. reported on the safety and efficacy of nivolumab among non-small cell lung cancer (NSCLC) patients, including those aged ≥70 years or with poor PS (CheckMate 153). In the CheckMate 153 trial, the levels of safety in the overall population and patients with PS 2 were almost the same (serious treatment adverse events: 5–6%). However, the median OS of the overall population was 9.1 months and that of patients with PS 2 was only 4.0 months [11]. Although, there were few studies of nivolumab in patients with poor PS (PS 2,3, and 4).

Mishima S et al. reported significant improvements in objective RR (ORR) and PFS among patients with PS 0 compared with those with PS 1 or 2 (ORR: 30% vs. 3%, *p* < 0.01; median PFS 3.0 vs.1.1 months; hazard ratio [HR] 0.30, 95% CI 0.18–0.52, *p* < 0.01) [12]. For other cancers such as NSCLC and malignant melanoma, several studies reported that poor PS was associated with poor survival outcome. Fujimoto D et al. reported that smoking status, *EGFR* mutation/*ALK* rearrangement and poor PS were independent poor prognostic factors among NSCLC patients in a multicentre retrospective cohort study (PS 0–1 vs 2–4; HR 0.41, *p* < 0.001) [13]. Katsura H et al. studied the efficacy and safety of nivolumab among NSCLC patients with poor PS. The OS durations of patients with PS 0–1 and 2–4 were 412 and 32 days, respectively (*p* < 0.001) [14]. Our study is the first to focus on nivolumab for AGC patients with poor PS. In our study, the OS among patients with poor PS was significantly shorter than that among those with good PS (83 vs. 177 days, *p* = 0.0255). The same trend was observed in our study.

In a previous study of NSCLC (CheckMate 153 trial), irAEs were similar for the overall population (6%) and patients with an ECOG PS of 2 (9%) [11]. Katsura H et al. reported that the incidence of pneumonitis in the group with poor PS was significantly higher than that in the group with good PS (35% vs. 9%, *p* = 0.028) [14]. Fujimoto D et al. reported that the incidence rates of severe irAEs were similar between those with good PS scores (0–1) and poor PS scores (2–4) within 2 months after commencing nivolumab therapy (6.1% vs. 6.3%, respectively; *p* = 0.918). However, 3 out of 4 patients who developed toxicities of grade 5 had poor PS [13]. In our study, there were similar frequencies of treatment-related adverse events between the Good and Poor groups, but 1 patient with poor PS developed grade 5 toxicity. These results suggested that severe toxicity needed to be noted in the Poor PS group.

In our study, patients with poor PS received limited benefit from nivolumab; only 1 patient with poor PS achieved PR due to complications such as brain infarction. Only 2 patients achieved a PFS of 6 months in the Poor group; the poor PS among both patients was due to brain infarction and osteoarthritis of the hip. In the Poor group, 20 patients recorded poor PS due to gastric cancer; the RR was 0% and the DCR was 5%. These results suggested that a high tumour burden was associated with poor outcome among gastric cancer patients who received nivolumab.

In AGC patients, no biomarker has been established to predict the efficacy of nivolumab therapy. We investigated whether JCOG index, RMH index and mGPS could be predictive factor for poor PS patients, but none could be a predictive factor. The reason may be that poor PS was already included as a factor in JCOG index and RMH index. In addition, only 4 cases had mGPS of 0 in poor PS patients. We consider the need for studies to validate new predictive factors of nivolumab efficacy in poor PS AGC patients.

## Conclusions

Our results indicate that nivolumab has a modest effect and is feasible as third- or later-line therapy for AGC patients. However, among patients with poor PS, this study suggested that the effect may be insufficient. This study suggested that nivolumab is not recommended for AGC patients with poor PS. The present study had several limitations, including its retrospective design, single institution setting, and small number of patients. Further studies to investigate useful predictive factor with larger numbers of patients are needed.

## Data Availability

All the data and materials supporting the conclusions were included in the main paper. The datasets used in the current study could be available from the corresponding author on request.

## Abbreviations

AGC: Advanced gastric cancer
ALP: Alkaline phosphatase
CRP: C-reactive protein
CTCAE: Common Terminology Criteria for Adverse Events
DCR: Disease control rate
ECOG: Eastern Cooperative Oncology Group
JCOG: Japan Clinical Oncology Group
NSCLC: Non-small cell lung cancer
OS: Overall survival
PFS: Progression-free survival
PS: Performance status
RECIST: Response Evaluation Criteria in Solid Tumors
RMH: Royal Marsden Hospital
RR: Response rate
